# Limited value of procalcitonin, C-reactive protein, white blood cell, and neutrophil in detecting bacterial coinfection and guiding antibiotic use among children with enterovirus infection

**DOI:** 10.1007/s12519-021-00504-2

**Published:** 2022-01-21

**Authors:** Rui-Mu Zhang, Kun Tan, Shu Fu, Ji-Kui Deng

**Affiliations:** Department of Infectious Diseases, Shenzhen Children’s Hospital, Shenzhen, 518038 China

Procalcitonin (PCT), C-reactive protein (CRP), and white blood cell (WBC) have been used as markers of bacterial infection in children for decades. Previous studies have suggested PCT, CRP, WBC, and percentage of neutrophils (%N) may be useful in detecting bacterial infection in children [1–4]. However, elevated levels of these biomarkers have also been noted in children with enterovirus infection [5–7]. In a study involving 5692 hospitalized children with herpangina or hand, foot, and mouth disease (HFMD) in two periods of years, the medians of CRP were 50.1 and 42.5 mg/L, respectively; and the medians of WBC were 14.1 and 15.3 × 10^9^/L, respectively [5]. These biomarkers were sometimes considered as evidence of bacterial coinfection in children with enterovirus infection, which resulted in a high antibiotic prescribing rate. For children hospitalized for HFMD, the antibiotic prescribing rates ranged from 7.4% to 100% in previous studies [5, 8, 9]. However, the value of these biomarkers in detecting bacterial coinfection among children with enterovirus infection is unclear.

We conducted a retrospective study in Shenzhen Children’s Hospital, a 1300-bed tertiary care facility in Shenzhen, China. The study population consisted of all children hospitalized for herpangina or HFMD between January 2015 and December 2020. Enterovirus infection was defined as the presence of a positive polymerase chain reaction (PCR) test for enterovirus with an oropharyngeal swab or stool specimens. Single enterovirus infection was defined as the presence of enterovirus infection which could fully explain all the symptoms of the patient. Enterovirus infection severity was classified as mild or severe based on the Chinese guideline for the diagnosis and treatment of HFMD (2018 edition) [10]. Definitions of bacterial coinfection diseases are summarized in Table 1. Cases were defined as patients with enteroviral and bacterial coinfection disease. Two controls with a single enterovirus infection were matched to each case by age (days) and sex. For cases who could not be matched by exactly the same age, they would be matched with controls of the most similar age.

**Table 1:** Definitions of bacterial coinfection diseases in children with enterovirus infection

Bacterial coinfection diseases	Definitions
Sepsis	Expert consensus for the diagnosis and management of septic shock (infectious shock) in children [11]
Bacterial pneumonia	Radiographic diagnosis of pneumonia + positive BALF/blood culture for bacteria
Bacterial enteritis	Diarrhea + positive culture/PCR test for bacteria from stool
Urinary tract infection	Fever/urinary symptoms + pyuria/positive urine culture for bacteria
Purulent tonsillitis	Tonsillar exudate + neutrophilia/positive RADT for GAS/positive throat culture for bacteria
Staphylococcal scalded skin syndrome	Classic cutaneous findings of SSSS + recovery after antibiotic treatment/positive culture for Staphylococcus aureus
Skin and soft tissue infections	Erythema, swelling, heat, and pain + recovery after antibiotic treatment/positive culture for bacteria

*BALF* bronchoalveolar lavage fluid; *PCR* polymerase chain reaction; *RADT*, rapid antigen detection test; *GAS*, group A *Streptococcus*; *SSSS* staphylococcal scalded skin syndrome

Patients with any of the following factors were excluded: negative or absence of PCR test for enterovirus; absence of both the CRP and PCT tests; comorbidity other than bacterial coinfection; liver dysfunction (prothrombin time > 18 seconds and serum bilirubin ≥ 20 μmol/L) [12]; immunocompromised state or immunodeficiency; underlying chronic disease (autoimmune disease, thyroid disease, malnutrition, congenital heart disease, and chronic lung disease).

The clinical variables were measured every day during hospitalization. Blood samples were collected during hospitalization as needed to guide management decisions. Categorical variables were presented as number and percentage. Continuous variables were presented as mean ± standard deviation (SD) if they were normally distributed or median (25–75% interquartile range) if they had a skewed distribution. Chi-square test was used for categorical variables. The Student *t* test or Mann–Whitney test was used for continuous variables, as appropriate. Binary logistic regression analysis was also performed to control confounding effects. Data analysis was performed by SPSS 26.0 software. All *P*-values were two-tailed, and *P* < 0.05 was considered to indicate statistical significance.

We identified 45 cases and 90 controls (Fig. 1, Table 2). CBC and CRP tests were performed in all the included children. PCT test was performed in 37 cases and 83 controls. The medians of test timing (days after fever onset) for PCT, CRP, WBC, and N% were, respectively, 4, 4, 3, 3 in cases and 3, 2, 2, 2 in controls. The maximal levels of inflammatory biomarkers in cases were as follows: PCT, 6.78 ng/mL; CRP, 135.1 mg/L; WBC, 32.53 × 109/L; and %N, 88.4%. The maximal levels of inflammatory biomarkers in controls were as follows: PCT, 6.91 ng/mL; CRP, 120 mg/L; WBC, 42.73 × 109/L; and %N, 88.3%. One case and three controls were infected by enterovirus 71 (EV71); one case and one control were infected by coxsackie A16 (CA16); and the remaining patients were infected by non-EV71 and non-CA16 enteroviruses. Of the 45 cases, 18 with bacterial enteritis had positive stool cultures for nontyphoidal *Salmonella*; one case with bacterial enteritis had positive blood and stool cultures for *Salmonella typhi*; five cases with urinary tract infection had positive urine cultures for bacteria; and the remaining cases had no positive culture results. No multiple bacterial infections were found in all cases. Most of the 45 cases received antibiotic treatment except for two cases with mild salmonella enteritis. None of the children were admitted to the intensive care unit and all of them were discharged with alleviation of symptoms.

**Fig. 1:**
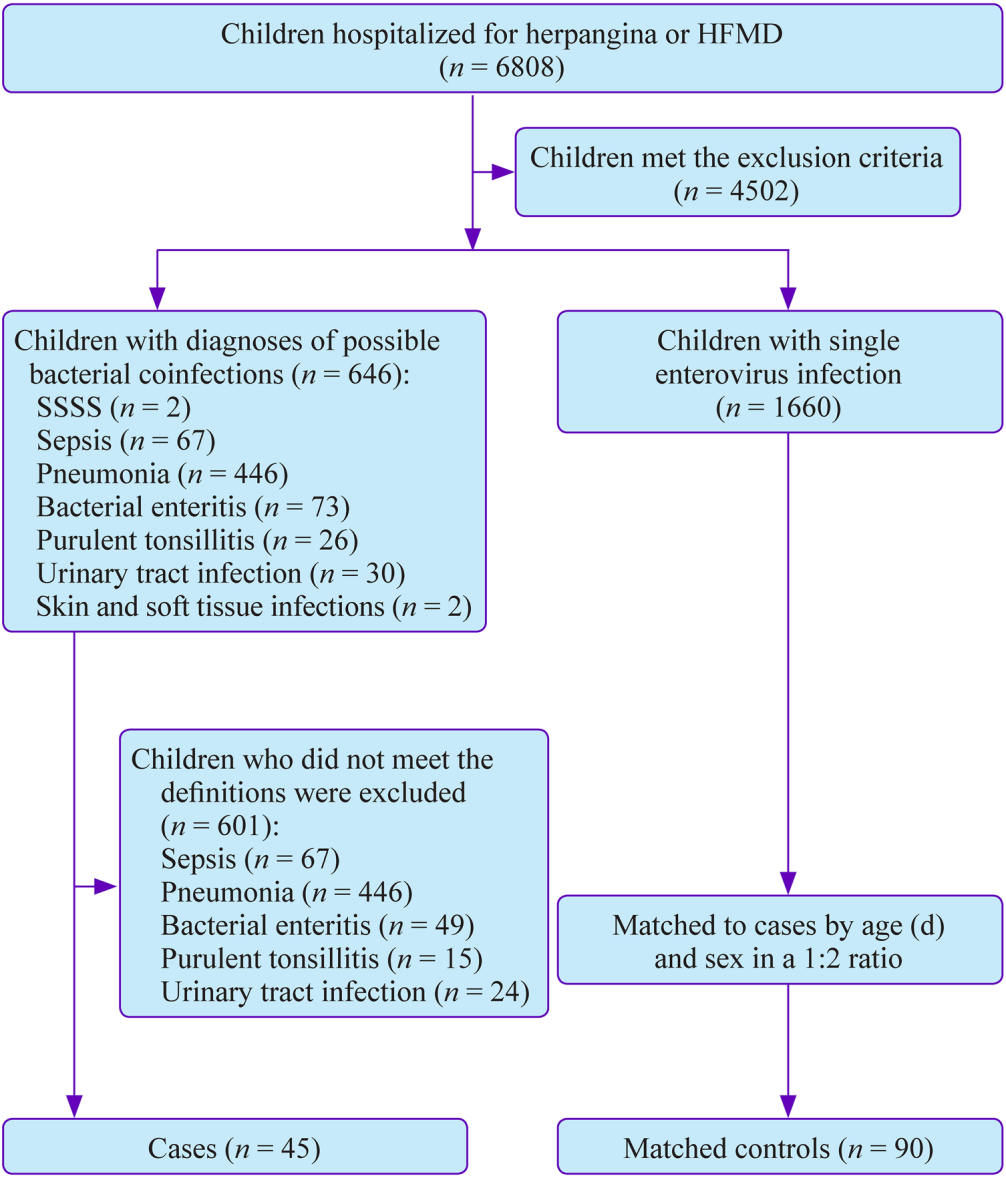
Flow diagram for study design and data collection. *HFMD* hand, foot, and mouth disease, *SSSS* staphylococcal scalded skin syndrome

**Table 2:** Characteristics of hospitalized children with enteroviral and bacterial coinfection (Cases) and children with single enterovirus infection (Controls)

Characteristics	Cases (*n* = 45)	Controls (*n* = 90)	*P* value
Demographics			
Male, *n* (%)	34 (76)	68 (76)	–
Female, *n* (%)	11 (24))	22 (24)	–
Age, d	537 (393–732)	536 (394–729)	0.966
Clinical features			
Herpangina, *n* (%)	11 (24)	14 (16)	0.210
Severe enterovirus infection, *n* (%)	23 (51)	39 (43)	0.393
Duration of fever, d	4 (3–7)	3 (2–4)	–
Antibiotic treatment, *n* (%)	43 (96)	49 (54)	–
Coinfection diseases, *n*			
SSSS	2	0	–
Skin and soft tissue infection	2	0	–
Urinary tract infection	6	0	–
Purulent tonsillitis	11	0	–
Bacterial enteritis	24	0	–
Biomarkers			
PCT, ng/mL^a^	0.21 (0.08–0.64)	0.10 (0.05–0.24)	0.012
CRP, mg/L	24.90 (11.35–54.20)	24.10 (9.43–47.78)	0.704
WBC count, × 10^9^/L	15.01 (11.18–16.92)	15.64 (13.25–20.73)	0.140
%N, %	64 (51–74)	68 (59–75)	0.317

Numbers are shown as median (25–75% interquartile range) unless otherwise indicated

*SSSS* staphylococcal scalded skin syndrome, *PCT* procalcitonin, *CRP *C-reactive protein, *WBC* white blood cell, *%N* percentage of neutrophils

^a^PCT test was performed in 37 cases and 83 controls

Mann–Whitney test indicated that PCT level [0.21 (0.08–0.64] vs. 0.10 (0.05–0.240 mg/L, *P* = 0.012)] was significantly higher in the cases than in the controls. In the further binary logistic regression analysis, there were no significant differences in PCT, CRP, WBC, or %N between the cases and the controls (Table 3).

**Table 3:** Multivariate analysis of biomarkers in hospitalized children with enteroviral and bacterial coinfection (cases) and children with single enterovirus infection (Controls)

Variables	Cases (*n* = 45)	Controls (*n* = 90)	*P* value	Odds ratio (95% CI)
PCT, ng/mL^a^	0.21 (0.08–0.64)	0.10 (0.05–0.24)	0.147	1.346 (0.901–2.011)
CRP, mg/L	24.90 (11.35–54.20)	24.10 (9.43–47.78)	0.417	1.006 (0.991–1.021)
WBC count, × 10^9^/L	15.01 (11.18–16.92)	15.64 (13.25–20.73)	0.140	0.945 (0.877–1.019)
%N, %	64 (51–74)	68 (59–75)	0.617	0.992 (0.961–1.024)
Herpangina, *n* (%)	11 (24)	14 (16)	0.150	2.132 (0.761–5.975)
Severe enterovirus infection, *n* (%)	23 (51)	39 (43)	0.371	1.478 (0.628–3.480)

Numbers are shown as median (25–75% interquartile range) unless otherwise indicated

*CI* Confidence interval; *PCT* procalcitonin; *CRP* C-reactive protein; *WBC* white blood cell; *%N* percentage of neutrophils

^a^PCT test was performed in 37 cases and 83 controls

Of the 90 controls, 49 (54%) received antibiotic treatment. Mann–Whitney test and chi-square test indicated that there were no significant differences in duration of fever [2.00 (2.00–3.00)] vs. 3.00 (2.00–4.00) days, *P* = 0.075] and length of hospitalization [ 4.00 (3.00–5.00) vs. 4.00 (3.00–4.00) days, *P* = 0.260] between controls with and without antibiotic treatment.

In this study, we identified 45 children with enteroviral and bacterial coinfection and 90 matched children with single enterovirus infection. Univariate and multivariate analysis suggested that there were no significant differences in the biomarkers between the two groups, revealing the poor utility of these biomarkers in identifying bacterial coinfection among children with enterovirus infection. Similarly, CRP, WBC, and %N also showed limited diagnostic value in fracture-related infections in adults [13]. This indicates the application of inflammatory biomarkers may not be suitable under certain circumstances, and the results should be taken with caution. We also found that antibiotics did not significantly shorten the duration of fever or length of hospitalization in children with a single enterovirus infection. Clinicians should not prescribe antibiotics only based on elevated levels of inflammatory biomarkers.

## Data Availability

The datasets generated during and/or analyzed during the current study are available from the corresponding author on reasonable request.
